# Early Immune Checkpoint Inhibitor Administration Increases the Risk of Radiation-Induced Pneumonitis in Patients with Stage III Unresectable NSCLC Undergoing Chemoradiotherapy

**DOI:** 10.3390/cancers17101711

**Published:** 2025-05-20

**Authors:** Yiwei Qin, You Mo, Pengwei Li, Xinyi Liang, Jinming Yu, Dawei Chen

**Affiliations:** 1 Department of Radiation Oncology, Cheeloo College of Medicine, Shandong University Cancer Center, Jinan 250012, China; qyw000728@163.com; 2 Department of Shandong Provincial Key Laboratory of Precision Oncology, Shandong Cancer Hospital and Institute, Shandong First Medical University (Shandong Academy of Medical Sciences), Jinan 250117, China; gemweiwei619495@163.com (P.L.); leungyanyee@163.com (X.L.); 3 Department of Cardiovascular Medicine, The First Affiliated Hospital of Shantou University Medical College, Shantou University, Shantou 515000, China; moyou.moyou@163.com; 4 School of Clinical Medicine, Shandong Second Medical University, Weifang 261000, China

**Keywords:** chemoradiotherapy, immunotherapy, immune checkpoint inhibitors, non-small cell lung cancer, radiation pneumonitis

## Abstract

Background/Objectives: The PACIFIC trial showed that immune checkpoint inhibitors (ICI) administered after concurrent chemoradiotherapy (cCRT) significantly improve survival in stage III unresectable non-small cell lung cancer (NSCLC). However, the optimal timing of ICI administration with cCRT is still debated, with concerns about increased risks of adverse effects, particularly radiation-induced pneumonitis (RP), from combining radiotherapy and immunotherapy. Methods: A search of multiple databases identified studies on stage III unresectable NSCLC patients receiving cCRT and ICI. A meta-analysis was performed utilizing the meta package in R software. Furthermore, data from 170 patients treated at Shandong Cancer Hospital and Institute between 2019 and 2023 were analyzed to assess RP following cCRT and ICI treatment. Results: The meta-analysis revealed that the incidences of ≥grade 2 RP were 25.3%, 24.3%, and 45.3% in the ICI following cCRT group, the ICI concurrent with cCRT group, and the ICI prior to cCRT group, respectively. The ICI prior to cCRT group exhibited significantly elevated rates. In the clinical retrospective study, ≥grade 2 RP was more prevalent in the ICI concurrent with cCRT group (HR: 2.258, 95% CI: 1.135–4.492, *p* = 0.020) and the ICI prior to cCRT group (HR: 2.843, 95% CI: 1.453–5.561, *p* = 0.002) compared with the ICI following cCRT group. Furthermore, a shorter interval between treatments correlates with an increased incidence of RP. Conclusions: Advancing the timing of ICI administration is associated with an increased incidence of ≥grade 2 RP following cCRT in patients with stage III unresectable NSCLC.

## Simple Summary

This study explores the timing of immune checkpoint inhibitors in patients with stage III unresectable non-small cell lung cancer who receive a combination of chemoradiotherapy and immune treatment. While previous research has shown that adding immunotherapy following chemoradiotherapy improves survival, concerns exist that initiating immunotherapy too early may increase the risk of severe lung damage. This study aims to determine the optimal timing for administering immunotherapy to minimize this risk. The findings suggest that initiating immunotherapy prior to or concurrent with chemoradiotherapy may significantly increase the risk of severe lung side effects, particularly pneumonitis. This research helps guide treatment strategies to improve patient outcomes while minimizing treatment-related adverse effects.

## 1. Introduction

Lung cancer remains the leading cause of cancer-related mortality globally. Non-small cell lung cancer (NSCLC) comprises approximately 80% of pulmonary malignancies, of which one-third present with stage III disease at diagnosis, characterized by locally advanced, unresectable progression. The PACIFIC trial [1] established durvalumab consolidation therapy as the standard of care following concurrent chemoradiotherapy (cCRT) for these patients.

Numerous clinical trials are exploring the benefits of combining radiotherapy (RT) with immunotherapy to improve treatment outcomes. Studies such as LUN14-179 [2], GEMSTONE-301 [3], RTOG 3505 [4], and the COAST study [5] examine immune checkpoint inhibitors (ICI) following cCRT, while studies including PACIFIC-2 [6], KEYNOTE-799 [7], and CheckMate-73L [8] investigate concurrent ICI administration. The AFT-16 study [9] focuses on ICI prior to cCRT. However, the optimal sequencing of ICI therapy with cCRT remains debated, and its safety requires further evaluation. The combination may lead to additive toxicity, posing challenges in developing effective and tolerable treatment plans.

Radioimmunotherapy carries an elevated risk of adverse events impacting multiple organ systems [10], most notably radiation-related pneumonitis (RP). RP is common and potentially fatal, characterized by interstitial pneumonia and pulmonary fibrosis, which significantly impacts patient quality of life and survival [11]. Furthermore, clinical observations suggest that patients receiving immunotherapy concurrent with or prior to cCRT experience more severe RP than those treated afterward.

Although several studies have explored RP in the context of ICI therapy, a significant gap remains in understanding the comparative effects of different ICI treatment sequences with cCRT in stage III unresectable NSCLC patients. This study aims to fill this gap by integrating meta-analysis and real-world retrospective research, thereby comparing the incidence of RP in patients undergoing ICI prior to, concurrent with, or following cCRT.

## 2. Materials and Methods

### 2.1. Meta-Analysis

#### 2.1.1. Search Strategy and Selection Criteria

This meta-analysis adheres to the PRISMA 2020 [12] and MOOSE guidelines [13] and has been registered with PROSPERO (CRD42024569310). Two authors independently searched PubMed, Embase, and Web of Science for eligible publications from 1 January 2010 to 30 July 2024 using specified search terms listed in Table A1. Related clinical trials were identified via ClinicalTrials.gov, and references were screened. Refer to Table A2**,** for detailed inclusion and exclusion criteria. For studies on similar populations, only the one with the most comprehensive data was included.

#### 2.1.2. Data Extraction

The following data were collected from eligible studies: basic information, such as the first author’s name, year of onset or consultation, and country of study; study stage and type; and NSCLC treatment details, including the ICI drug name, method of ICI integration with cCRT, and RP occurrence.

#### 2.1.3. Quality Assessment

The Cochrane Collaboration’s ‘risk of bias’ tool was utilized to evaluate and assess the quality of randomized controlled trials (RCTs) included in the meta-analyses. Real-world retrospective studies (RWSs) utilized the Newcastle–Ottawa Scale to assess study quality. Quality assessments were conducted by two independent authors with disagreements resolved by consensus and guidance from a senior author.

#### 2.1.4. Statistics Analysis

We assessed the incidence of ≥grade 2 and ≥grade 3 RP using SPSS (version 25.0) to calculate odds ratios (OR) and 95% confidence intervals (CI). A meta-analysis was conducted using the meta package in R software (version 4.4.1) to evaluate the toxicity of various cCRT and ICI regimens in stage III NSCLC, focusing on RP incidence and its 95% CI. The *I*^2^ statistic was applied to assess heterogeneity. Studies were considered heterogeneous if the chi-square test value was less than 0.1 or *I*^2^ exceeded 50%. In cases of heterogeneity, a random effects model was utilized to attenuate its impact on the results.

### 2.2. Real-World Retrospective Study

#### 2.2.1. Study Design and Patients

This retrospective analysis encompassed patients treated with cCRT and ICI at Shandong Cancer Hospital and Institute between March 2019 and December 2023. The study focused on patients with stage III NSCLC who received concurrent cCRT and ICI. Refer to Table A3 for detailed inclusion and exclusion criteria for patients. This study was approved by the institutional review board of Shandong Cancer Hospital and Institute and conducted in accordance with the Declaration of Helsinki.

#### 2.2.2. Treatment Strategy

**Figure 1:**
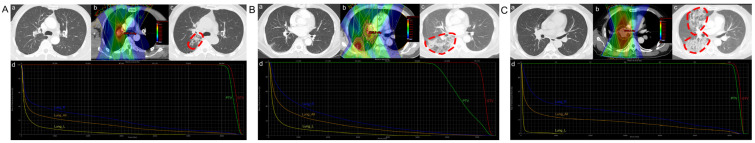
Typical clinical cases of radiation-related pneumonitis. (**A**) ICI following cCRT. (**B**) ICI concurrent with cCRT. (**C**) ICI prior to cCRT. (**a**) CT scan obtained before RT. (**b**) Isodose curve for the treatment plan. (**c**) CT scan obtained 3 months after RT. (**d**) Dose distribution histograms for the total lung, right lung, and left lung. The region where the red dashed circle on the CT images indicates radiation-related pneumonitis developed. Abbreviations: ICI, immune checkpoint inhibitors; cCRT, concurrent chemoradiotherapy; CT, computed tomography; RT, radiotherapy.

Treatment data for patients were retrieved from the medical records system. All patients received intensity-modulated RT with a total dose of ≥40 Gy, in conjunction with concurrent platinum-based chemotherapy. Furthermore, all patients underwent ICI therapy. ICI therapy entailed the administration of anti-programmed death 1 (anti-PD1) or anti-programmed death-ligand 1 (anti-PD-L1), administered prior to, concurrent with, or following cCRT. ICI prior to cCRT refers to the administration of ICI before the initiation of cCRT. ICI concurrent with cCRT involves the simultaneous administration of immunotherapy with or during RT. ICI following cCRT refers to the initiation of immunotherapy after completing the entire course of RT. Representative computed tomography (CT) images of affected patients are displayed in Figure 1.

#### 2.2.3. Diagnosis and Classification of RP

This study investigated patients’ clinical manifestations, imaging findings, therapeutic interventions, and clinical outcomes to assess the severity of RP. Subsequent CT scans were independently evaluated by two experienced radiologists. Any discrepancies were resolved through collaboration with a third expert radiologist. RP severity was graded on a scale from 1 to 5 according to the Common Terminology Criteria for Adverse Events v5.0 [14].

#### 2.2.4. Statistical Analysis

Continuous variables were reported as mean ± standard deviation (SD) or median (interquartile range, IQR), while categorical variables were represented as counts. Statistical analyses comprised one-way Analysis of Variance and Chi-Square Test or Fisher’s Exact Test for categorical variables. Kaplan–Meier curves were utilized to evaluate RP occurrence, with hazard ratios (HR) and 95% CI computed via multivariable Cox regression. All tests were two-tailed, with significance defined as *p* < 0.05. Analyses were performed using SPSS (version 25.0), while data visualization was conducted using GraphPad Prism (version 9.5) and R (version 4.4.1).

## 3. Results

### 3.1. Results in Meta-Analysis

#### 3.1.1. Search Results

Initially, 1761 records were retrieved through a comprehensive database search. Following duplicate removal, 1245 records were retained. After reviewing the titles and abstracts, 1148 records were excluded, leaving 97 articles for full-text evaluation. Ultimately, 28 studies, comprising 3576 patients, were deemed eligible for inclusion. Of these, 10 were single-arm studies (2 RCTs and 8 RWSs), whereas 18 were two-arm studies (9 RCTs and 9 RWSs). The study selection process and the reasons for exclusion are depicted in Figure S1.

#### 3.1.2. Characteristics and Methodological Quality of the Studies

This meta-analysis comprised 28 studies. Of these, 19 studies involved 2695 patients receiving ICI prior to cCRT [1,2,4,5,15,16,17,18,19,20,21,22,23,24,25,26,27,28,29], 6 studies involved 568 patients receiving ICI concurrent with cCRT [7,29,30,31,32,33], and 4 studies involved 313 patients receiving ICI following cCRT [9,34,35,36]. Additionally, one RWS with two arms was included in both the ICI following cCRT and ICI concurrent with cCRT groups [29]. Of the 28 studies, 15 were prospective, while 13 were retrospective. All studies investigating cCRT and ICI were published after 2017. The characteristics of the included studies are outlined in Table 1. The quality assessment of all two-arm RCTs is depicted in Figure S2. The quality of the two-arm non-RCTs was assessed using the NOS criteria, with the results provided in Table A4.

#### 3.1.3. Results of the Statistical Analyses

Most single-arm studies exhibited substantial variability, as demonstrated by a chi-square test value of *p* < 0.1 or *I*^2^ > 50%; consequently, a random effects model was utilized to minimize its influence on the results. Both single-arm and two-arm studies were analyzed to evaluate the effect of varying cCRT and ICI treatment sequences on RP incidence.

**Figure 2:**
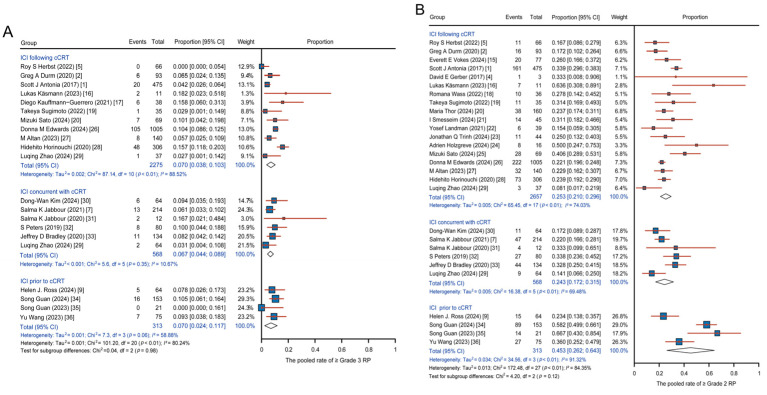
Pooled analysis of RP across single-arm studies with various regimens. (**A**) Pooled incidence of ≥grade 3 RP. (**B**) Pooled incidence of ≥grade 2 RP. Abbreviations: ICI, immune checkpoint inhibitors; cCRT, concurrent chemoradiotherapy; RP, radiation-related pneumonitis.

No significant difference was observed in the pooled incidence of ≥grade 3 RP among the three groups (0.070, 95% CI: 0.038–0.103; 0.067, 95% CI: 0.044–0.089; and 0.070, 95% CI: 0.024–0.117 for ICI following cCRT, ICI concurrent with cCRT, and ICI prior to cCRT, respectively) (Figure 2A). For the incidence of ≥grade 2 RP, the pooled incidence was 0.253 (95% CI: 0.210–0.296) in the ICI following cCRT group, 0.243 (95% CI: 0.172–0.315) in the ICI concurrent with cCRT group, and 0.453 (95% CI: 0.262–0.643) in the ICI prior to cCRT group. The pooled incidence of ≥grade 2 RP was higher in the ICI prior to cCRT group (Figure 2B). Due to the significant heterogeneity, further sensitivity analysis was performed for the incidence of ≥grade 2 RP, and the results are shown in Figure S3. Logistic regression analysis revealed no significant difference in the occurrence of ≥grade 3 RP among the three groups (Figure S4A). Specifically, the ICI prior to cCRT group exhibited a higher likelihood of ≥grade 2 RP compared to the other two groups, with OR of 2.549 (95% CI: 2.008–3.237, *p* < 0.001) compared to ICI following cCRT and 2.589 (95% CI: 1.933–3.468, *p* < 0.001) compared to ICI concurrent with cCRT (Figure S4B). These findings suggest that administering ICI prior to cCRT increases the incidence of RP.

### 3.2. Results in Real-World Retrospective Study

#### 3.2.1. Patient Characteristics

Our findings are corroborated by real-world data. This study comprised 170 patients. Demographic and treatment characteristics of the patients are outlined in Table 2. Of the 170 patients, 117 (68.8%) received ICI consolidation after cCRT (ICI following cCRT group), 23 (13.5%) received cCRT concurrently with ICI (ICI concurrent with cCRT group), and 30 (17.7%) received cCRT following ICI induction (ICI prior to cCRT group). The median age of the patients was 61 years (IQR, 55–68). The majority of patients were male (92.4%) and smokers (69.4%). Furthermore, 68.8% had squamous cell carcinoma, 38.2% had stage IIIA, 48.8% had stage IIIB, and 12.9% had stage IIIC. Moreover, 64.1% of tumors were located in the upper lobes of the lungs, with 48.8% in the left lung. RT was delivered using intensity-modulated radiation therapy, with a median single dose of 2 Gy (IQR, 2, 2) and a median total dose of 60 Gy (IQR, 56, 60). Among the patients, 66.5% received PD-1 inhibitors (including tislelizumab, nivolumab, sintilimab, pembrolizumab, camrelizumab, toripalimab, penpulimab, and serplulimab), whereas 33.5% received PD-L1 inhibitors (including durvalumab, sugemalimab, and atezolizumab).

#### 3.2.2. Radiation-Related Pneumonia Outcome

**Figure 3:**
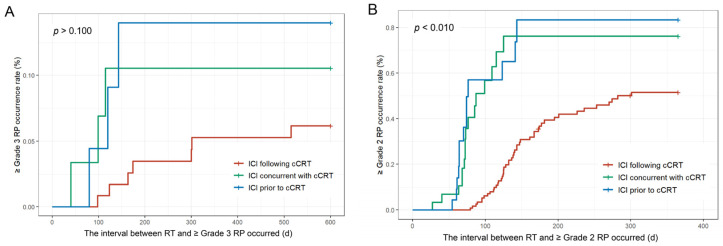
Kaplan–Meier survival curves for patients with RP. (**A**) Incidence of ≥grade 3 RP. (**B**) Incidence of ≥grade 2 RP. Abbreviations: ICI, immune checkpoint inhibitors; cCRT, concurrent chemoradiotherapy; RT, radiotherapy; RP, radiation-related pneumonitis.

As shown in Table 2, among the 170 patients, 76 (44.7%) experienced ≥grade 2 RP, and 13 (7.6%) developed ≥grade 3 RP. Specifically, in the ICI following cCRT group (117 patients), 47 (40.1%) experienced ≥grade 2 RP, and 7 (5.9%) developed ≥grade 3 RP. In the ICI concurrent with cCRT group (23 patients), 13 (56.5%) developed ≥grade 2 RP, while 3 (13.0%) developed ≥grade 3 RP. In the ICI prior to cCRT group (30 patients), 16 (53.3%) developed ≥grade 2 RP, while 3 (10.0%) developed ≥grade 3 RP. Kaplan–Meier curves indicated that, compared to the ICI following cCRT group, there was no statistically significant difference in the incidence of ≥grade 3 RP among the three groups (*p* > 0.100) (Figure 3A). Moreover, the ICI concurrent with cCRT group and the ICI prior to cCRT group were more likely to develop ≥grade 2 RP (*p* < 0.010) (Figure 3B), and did so at an earlier time (Figure S4). Among the 37 patients who received durvalumab consolidation following cCRT, 16 (43.2%) developed ≥grade 2 RP, and 3 (8.1%) developed ≥grade 3 RP, offering real-world data on the PACIFIC regimen in China.

**Figure 4:**
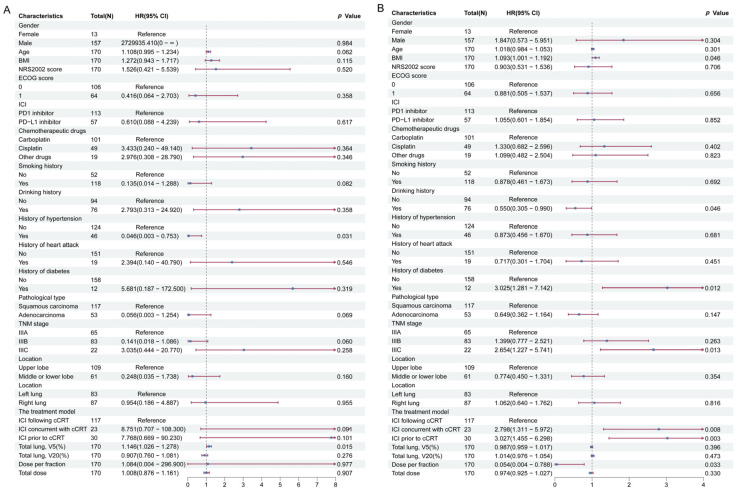
Cox regression analysis associated with RP. (**A**) Risk factors for ≥grade 3 RP. (**B**) Risk factors for ≥grade 2 RP. Abbreviations: HR, hazard ratios; CI, confidence intervals; ICI, immune checkpoint inhibitors; cCRT, concurrent chemoradiotherapy; RP, radiation-related pneumonitis.

Variables such as gender, age, and BMI were included in the multifactorial Cox regression analysis to identify risk factors for RP, as presented in Figure 4. The results suggest no statistically significant difference in the occurrence of ≥grade 3 RP among the three groups (Figure 4A). Notably, the ICI concurrent with cCRT group (HR = 2.798; 95% CI: 1.311–5.972; *p* = 0.008) and the ICI prior to cCRT group (HR = 3.027; 95% CI: 1.455–6.298; *p* = 0.003) were more likely to develop ≥grade 2 RP compared to the ICI following cCRT group (Figure 4B). These real-world findings align with the results of the meta-analysis.

#### 3.2.3. Interval Between ICI and RT Relating to RP

**Figure 5:**
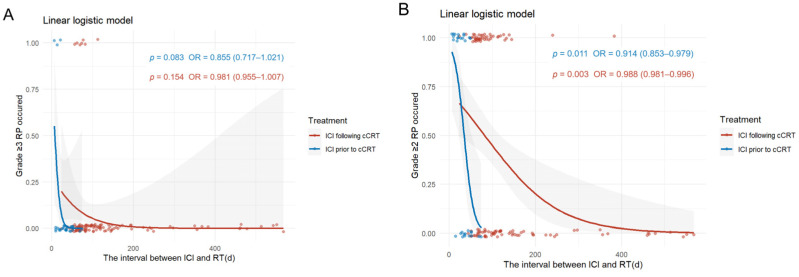
Relationship between the interval between RT and ICI and the occurrence of RP. (**A**) Incidence of ≥grade 3 RP. (**B**) Incidence of ≥grade 2 RP. Abbreviations: ICI, immune checkpoint inhibitors; cCRT, concurrent chemoradiotherapy; RT, radiotherapy; RP, radiation-related pneumonitis.

In the ICI prior to cCRT group, the interval between ICI and RT was defined as the difference between the last use of ICI during the induction immunotherapy phase and the start of RT. A longer interval was associated with a reduced likelihood of developing ≥grade 2 RP (OR = 0.914; 95% CI: 0.953–0.979; *p* = 0.011). Similarly, in the ICI following cCRT group, the interval between ICI and RT was defined as the difference between the first use of ICI during the consolidation phase and the start of RT. In this case, a longer interval was also associated with a lower risk of developing ≥grade 2 RP (OR = 0.988; 95% CI: 0.981–0.996; *p* = 0.003). Additionally, the incidence of ≥grade 2 RP rose more rapidly with shorter intervals in the ICI prior to cCRT group compared to the ICI following cCRT group (Figure 5A). The probability and rate of occurrence of ≥grade 3 RP exhibited a similar trend in both groups (Figure 5B). Furthermore, in the ICI prior to cCRT group, the median interval between ICI and RT for ≥grade 2 RP was 21 days (IQR, 10.75, 34); in the ICI following cCRT group, the median interval was 73 days (IQR, 62, 97) (Figure S5).

## 4. Discussion

To our knowledge, no studies have directly compared the effects of different cCRT and ICI sequencing on RP in stage III unresectable NSCLC. Our findings show that administering ICI prior to cCRT significantly increases RP incidence, a comparison not previously addressed in meta-analyses or clinical trials. The 2024 National Comprehensive Cancer Network guidelines recommend cCRT followed by durvalumab consolidation for stage III unresectable NSCLC, based on clinical trial data and US Food and Drug Administration approval [37]. As new sequencing regimens emerge, the relative safety—especially regarding RP—remains crucial. Our study may inform future randomized trials and clinical guidelines.

The meta-analysis revealed significant heterogeneity, likely due to variations in treatment protocols (ICI/cCRT drug selection, dosing, and scheduling), diverse study populations (ethnicity, age, comorbidities), and inconsistent RP grading criteria. Limited studies and small sample sizes in the “ICI concurrent with cCRT” and “ICI prior to cCRT” groups, combined with inter-study differences in patient characteristics and outcome assessments, restricted subgroup analyses. Sensitivity analysis showed stable ≥grade 2 RP rates in the “ICI following cCRT” and “ICI concurrent with cCRT” groups but marked variability in the “ICI prior to cCRT” group, indicating higher heterogeneity. Despite this, the “ICI prior to cCRT” group consistently exhibited higher ≥ grade 2 RP incidence than the “ICI following cCRT” group, aligning with the primary conclusion. Further research on these regimens is needed to refine subgroup analyses and reduce heterogeneity.

In this real-world study, we applied Minimal Clinically Important Difference (MCID) thresholds (Horita et al. [38]) to contextualize hazard ratios (HRs): Cohen’s d = 0.5 (HR = 1.56/0.64), d = 0.3 (HR 1.31/0.76), and d = 0.2 (HR = 1.20/0.83). The observed HRs for ≥grade 2 RP—2.258 (ICI concurrent with cCRT) and 2.843 (ICI prior to cCRT)—exceeded these thresholds, confirming both statistical and clinical significance. Notably, ≥grade 3 RP risks were elevated in ICI concurrent cCRT (HR = 1.866, 95% CI 0.385–9.045) and ICI prior to cCRT (HR = 3.075, 95% CI 0.531–17.812) groups compared to ICI following cCRT (both *p* > 0.2), with effect sizes surpassing the MCID threshold (HR > 1.56), aligning with ≥grade 2 RP trends. Therefore, its potential clinical implications warrant close monitoring in patient management, particularly for high-risk populations, including those with pre-existing interstitial lung disease, chronic obstructive pulmonary disease, or advanced age [39,40,41]. These findings suggest that concurrent or prior ICI administration may potentially increase the clinical risk of ≥grade 3 RP, and the absence of statistical significance in the Cox regression analysis may stem from the sample size, which constrained statistical power and resulted in wide confidence intervals [42]. Proactive pulmonary function tests, early imaging, and risk–benefit evaluation of ICI timing with cCRT are recommended to mitigate potential severe RP risks.

Our findings suggest that a shorter interval between ICI and RT increases the likelihood of RP. Clinical outcomes of durvalumab after RT, presented at the 2018 European Society for Medical Oncology meeting, showed that administering durvalumab within 14 days post-RT improved survival outcomes. However, the rate of grade 3/4 adverse events was similar in patients receiving durvalumab ≥14 days or <14 days post-RT [43]. A retrospective study found that patients receiving ICI ≥21 days after stereotactic body radiotherapy had longer overall survival compared to those receiving it <21 days post-treatment [44]. These findings underscore the need for further investigation into the optimal interval between ICI and RT. Further studies are needed to determine the optimal dose, sequence, target area, and associated toxicity. Retrospective studies suggest that macrofractionated radiotherapy provides good local control, respectable survival, and manageable toxicity in older or less physically advanced patients with locally advanced NSCLC [45]. Standard multifraction radiotherapy may be associated with a lower likelihood of RP [46]. Should follow-up studies confirm that ICI improves survival but increases RP incidence, subsequent standard multifraction radiotherapy may be considered. Given the uncertainty surrounding the optimal timing and sequence of RT and ICI, patients should receive individualized treatment following multidisciplinary consultation.

Multiple meta-analyses and clinical studies have shown that administering ICI prior to cCRT significantly increases the risk of RP. This increased risk can be mechanistically attributed to three interconnected pathways: First, ICIs block the PD-1/PD-L1 axis, amplifying CD8^+^ T cell-mediated inflammation. This triggers the release of interferon-gamma and TNF-alpha, activating macrophages and promoting the transition of fibroblasts to myofibroblasts via TGF-β signaling [47,48]. Second, single-cell RNA sequencing has confirmed that RT and ICI cause the accumulation of senescent-like cells, creating a microenvironment prone to cytokine storms [49]. Third, lung microbiota dysbiosis and endocrine disorders (e.g., hypothyroidism) induced by ICIs may predispose pulmonary tissue to exaggerated inflammatory responses upon subsequent radiation exposure [50,51].

Regarding the pharmacological management of RP, the conventional use of glucocorticoids and antibiotics requires re-evaluation due to their paradoxical effects. Although Chinese consensus guidelines recommend these treatments for ≥grade 2 RP [52], emerging evidence suggests glucocorticoids worsen clinical outcomes in advanced NSCLC patients receiving ICI treatment [53], while antibiotics are linked to poorer outcomes in lung cancer patients [54]. To ensure an optimal balance between patient quality of life and survival, caution must be exercised in medication use, with prompt discontinuation if adverse effects occur [52].

This study has several limitations. First, there were few RCTs and RWSs directly comparing ICI use in stage III unresectable NSCLC patients prior to, concurrent with, or following cCRT. As a result, our meta-analysis relied partially on single-arm studies, which exhibited higher heterogeneity, limiting the strength of our conclusions. Second, the predominance of Chinese populations may affect the generalizability of our findings. However, our previous meta-analysis, which reviewed several databases, reflects the high incidence of lung cancer in East Asia, particularly China [55]. Additionally, a 2023 meta-analysis by Liu T et al. found comparable rates of severe toxicity (≥grade 3 RP) between Asian and non-Asian populations, suggesting that severe toxicity profiles may be consistent across ethnicities [56]. Moreover, the limited number of studies precludes effective subgroup analyses, increasing potential design bias and limiting the extrapolation of toxicity patterns to other ethnic populations. Additionally, inconsistencies in pneumonia grading criteria across studies may have biased the collection and reporting of RP. Furthermore, variations in cCRT and ICI regimens, RT duration, and dosage may have heightened the risk of intervention bias. Lastly, the patients were from a single institution, and the sample size was relatively small. Specifically, the ICI prior to cCRT group and the ICI concurrent with cCRT group consisted solely of patients recruited in prospective clinical trials, further limiting the number of eligible participants and potentially reducing the reliability of the findings compared to multicenter studies.

Despite these limitations, our study showed that immunotherapy sequencing impacts the incidence of ≥grade 2 RP and exacerbates toxic responses. Notably, patients in clinical trials may be healthier than those in real-world settings, suggesting that RP incidence may be higher in practice. Therefore, the safety of ICI and cCRT in stage III unresectable NSCLC patients requires careful consideration. In conclusion, future RCTs should focus on RP occurrence following changes in ICI sequencing and implement timely management strategies to improve survival. This study provides evidence for the safety of cCRT and ICI timing in stage III unresectable NSCLC patients, potentially informing future RCT design and clinical practice. We hope future RCTs will validate and refine our findings.

## 5. Conclusions

Earlier administration of ICI is associated with a significantly increased incidence of ≥grade 2 RP following cCRT in patients with stage III unresectable NSCLC. These findings emphasize the necessity of enhanced pulmonary monitoring during ICI therapy for stage III unresectable NSCLC. Furthermore, these results provide crucial guidance for future RCT design.

## Figures and Tables

**Table 1:** Characteristics of studies for single group rate meta-analysis.

Study	Author (Year)	Phase	Study Period	Country	Type of Study	Group	Treatment	Evaluated Patients (n)	≥Grade 2 RP (n)	≥Grade 3 RP (n)
COAST (NCT03822351)	Roy S Herbst (2022) [5]	II	2018–2020	Multicenter	Prospective	ICI following cCRT	Durvalumab following cCRT	66	11	0
HCRN LUN 14-179 *	Greg A Durm (2020) [2]	II	2015–2019	USA	Prospective	ICI following cCRT	Pembrolizumab following cCRT	93	16	6
NCT03840902	Everett E Vokes (2024) [15]	II	2019–2021	Multicenter	Prospective	ICI following cCRT	Durvalumab following cCRT	77	20	-
PACIFIC (NCT02125461)	Scott J Antonia (2017) [1]	III	2014–2016	Multicenter	Prospective	ICI following cCRT	Durvalumab following cCRT	475	161	20
RTOG 3505 (NCT02768558)	David E Gerber (2017) [4]	III	2016–2020	USA	Prospective	ICI following cCRT	Nivolumab following cCRT	3	1	-
RWS	Lukas Käsmann (2023) [16]		2016–2020	Germany	Prospective	ICI following cCRT	Nivolumab following cCRT	11	7	2
RWS	Diego Kauffmann-Guerrero (2021) [17]		NA	Germany	Prospective	ICI following cCRT	ICI following cCRT	38	-	6
RWS	Romana Wass (2022) [18]		NA	Austria	Prospective	ICI following cCRT	Durvalumab following cCRT	36	10	-
RWS	Takeya Sugimoto (2022) [19]		2019–2019	Japan	Prospective	ICI following cCRT	Durvalumab following cCRT	35	11	1
RWS	Maria Thor (2024) [20]		2017–2021	USA	Retrospective	ICI following cCRT	Durvalumab following cCRT	160	38	-
RWS	I Smesseim (2024) [21]		2018–2021	Netherlands	Retrospective	ICI following cCRT	Durvalumab following cCRT	45	14	-
RWS	Yosef Landman (2021) [22]		2018–2020	Israel	Retrospective	ICI following cCRT	Durvalumab following cCRT	39	6	-
RWS	Jonathan Q Trinh (2024) [23]		2012–2022	USA	Retrospective	ICI following cCRT	Durvalumab following cCRT	44	11	-
RWS	Adrien Holzgreve (2024) [24]		NA	Germany	Retrospective	ICI following cCRT	Durvalumab following cCRT	16	8	-
RWS	Mizuki Sato (2024) [25]		2013–2022	Japan	Retrospective	ICI following cCRT	Durvalumab following cCRT	69	28	7
RWS	Donna M Edwards (2024) [26]		2015–2021	USA	Retrospective	ICI following cCRT	Durvalumab following cCRT	1005	222	105
RWS	M Altan (2023) [27]		2018–2021	USA	Retrospective	ICI following cCRT	Durvalumab following cCRT	140	32	8
RWS	Hidehito Horinouchi (2020) [28]		2013–2015	Japan	Retrospective	ICI following cCRT	Durvalumab following cCRT	306	73	48
RWS	Luqing Zhao (2024) [29]		2013–2023	China	Retrospective	ICI following cCRT	ICI following cCRT	37	3	1
CLOVER study (NCT03509012)	Dong-Wan Kim (2024) [30]	I	2018–2020	Multicenter	Prospective	ICI concurrent with cCRT	Durvalumab concurrent with cCRT	64	11	6
KEYNOTE-799 (NCT03631784)	Salma K Jabbour (2021) [7]	II	2018–2020	Multicenter	Prospective	ICI concurrent with cCRT	Pembrolizumab concurrent with cCRT	214	47	13
NCT02621398	Salma K Jabbour (2020) [31]	I	2016–2018	USA	Prospective	ICI concurrent with cCRT	Pembrolizumab concurrent with cCRT	12	4	2
NICOLAS (NCT02434081)	S Peters (2019) [32]	III	2016–2018	Multicenter	Prospective	ICI concurrent with cCRT	Nivolumab concurrent with cCRT	80	27	8
RTOG 0617 (NCT00533949)	Jeffrey D Bradley (2020) [33]	III	2007–2013	Multicenter	Prospective	ICI concurrent with cCRT	Cetuximab concurrent with cCRT	134	44	11
RWS	Luqing Zhao (2024) [29]		2013–2023	China	Retrospective	ICI concurrent with cCRT	ICI concurrent with cCRT	64	9	2
AFT-16 (NCT03102242)	Helen J. Ross (2024) [9]	II	2017–2019	USA	Prospective	ICI prior to cCRT	Atezolizumab prior to cCRT	64	15	5
RWS	Song Guan (2024) [34]		2018–2022	China	Retrospective	ICI prior to cCRT	ICI prior to cCRT	153	89	16
RWS	Song Guan (2023) [35]		2018–2021	China	Retrospective	ICI prior to cCRT	ICI prior to cCRT	21	14	0
RWS	Yu Wang (2023) [36]		2018–2020	China	Retrospective	ICI prior to cCRT	ICI prior to cCRT	75	27	7

* The full title of HCRN LUN 14-179 is Hoosier Cancer Research Network LUN 14-179. Abbreviations: n, number; ICI, immune checkpoint inhibitors; cCRT, concurrent chemoradiotherapy; RWS, real-world retrospective study.

**Table 2:** Clinical and demographic characteristics by subgroup.

Characteristics	Total (n = 170)	ICI Following cCRT (n = 117)	ICI Concurrent with cCRT (n = 23)	ICI Prior to cCRT (n = 30)	*p* Value
Gender (n)					0.746
Female	13	10	1	2	
Male	157	107	22	28	
Age (median, IQR) (y)	61.0 (55.0, 68.0)	61.0 (55.5, 67.0)	62.0 (57.0, 69.0)	62.0 (53.8, 69.0)	0.810
BMI (mean ± SD) (kg/m^2^)	24.53 ± 3.04	24.48 ± 3.01	24.57 ± 2.80	24.69 ± 3.40	0.946
Systolic pressure (median, IQR) (mmHg)	125 (120, 134)	125 (121, 133.5)	130 (122, 135)	124 (115, 135)	0.352
Diastolic pressure (median, IQR) (mmHg)	77 (73.75, 84)	77 (73, 84)	78 (74, 87)	76 (72.75, 86.5)	0.913
NRS2002 score (median, IQR)	1 (1, 2)	1 (1, 2)	1 (1, 2)	1 (1, 2)	0.352
ECOG PS (n)					0.148
0	106	77	15	14	
1	64	40	8	16	
ICI (n)					<0.001
PD1 inhibitors	113	68	15	30	
PD-L1 inhibitors	57	49	8	0	
Smoking history (n)					0.868
No	52	37	6	9	
Yes	118	80	17	21	
Drinking history (n)					0.673
No	94	65	11	18	
Yes	76	52	12	12	
History of hypertension (n)					0.515
No	124	83	19	22	
Yes	46	34	4	8	
History of heart attack (n)					0.250
No	151	101	22	28	
Yes	19	16	1	2	
History of diabetes (n)					0.626
No	158	108	21	29	
Yes	12	9	2	1	
Pathological type (n)					0.009
Squamous carcinoma	117	72	20	25	
Adenocarcinoma	53	45	3	5	
TNM stage (n)					0.381
IIIA	65	40	9	16	
IIIB	83	62	11	10	
IIIC	22	15	3	4	
Location (n)					0.627
Upper lobe	109	76	16	17	
Middle and lower lobe	61	41	7	13	
Location, left or right lungs (n)					0.335
Left lung	83	53	12	18	
Right lung	87	64	11	12	
≥Grade 2 RP (n)					0.207
No	94	70	10	14	
Yes	76	47	13	16	
≥Grade 3 RP (n)					0.445
No	157	110	20	27	
Yes	13	7	3	3	
Dose per fraction (median, IQR) (Gy)	2 (2, 2)	2 (2, 2)	2 (2, 2)	2 (2, 2)	0.444
Total dose (median, IQR) (Gy)	60 (56, 60)	60 (56, 60)	60 (60, 60)	60 (54, 60)	0.290

Abbreviations: ICI, immune checkpoint inhibitors; cCRT, concurrent chemoradiotherapy; n, number; IQR, interquartile range; BMI, body mass index; SD, standard deviation; NRS2002, Nutritional Risk Screening 2002; ECOG PS, Eastern Cooperative Oncology Group Performance Status; PD-1, programmed death 1; PD-L1, programmed death-ligand 1; TNM stage, Tumor, Node, Metastasis Staging System; RP, radiation-induced pneumonitis.

## Data Availability

The datasets used and/or analyzed during the current study are available from the corresponding author upon reasonable request.

## Supplementary Materials

The following supporting information can be downloaded at: https://www.mdpi.com/article/10.3390/cancers17101711/s1, Figure S1: Literature selection and organization from multiple sources, illustrating the flowchart process; Figure S2: Quality assessment of the included RCTs; Figure S3: Sensitivity analysis of meta-analysis for ≥grade 2 RP; Figure S4: Comparing RP from different treatments using logistic regression; Figure S5: The interval between ICI and RT with ≥grade 2 RP.

## Abbreviations

The following abbreviations are used in this manuscript:

cCRT	Concurrent chemoradiotherapy
CI	Confidence intervals
CT	Computed tomography
HR	Hazard ratios
ICI	Immune checkpoint inhibitors
IQR	Interquartile range
NSCLC	Non-small cell lung cancer
OR	Odds ratios
PD-1	Programmed death 1
PD-L1	Programmed death-ligand 1
RCTs	Randomized controlled trials
RP	Radiation-related pneumonitis
RT	Radiotherapy
RWSs	Real-world retrospective studies
SD	Standard deviation

## Appendix A

**Table A1:** Search strategy.

**(a) Search strategy in PubMed**	
#	Query
#1	“Carcinoma, Non-Small-Cell Lung”[Mesh]
#2	((((((((Non Small Cell Lung) OR Non-Small-Cell Lung) OR Non-Small-Cell Lung Carcinomas) OR Non-Small Cell Lung Cancer) OR Non-Small-Cell Lung Carcinoma) OR Non Small Cell Lung Carcinoma) OR Nonsmall Cell Lung Cancer) OR Non-Small Cell Lung Carcinoma)
#3	#1 OR #2
#4	“Immunotherapy”[Mesh]
#5	(“Immunotherapy”[Mesh]) OR (Immunotherapies[Title/Abstract])
#6	“Immune Checkpoint Inhibitors”[Mesh]
#7	((((((((((((((((((Immune Checkpoint Blockers[Title/Abstract]) OR Immune Checkpoint Inhibitor[Title/Abstract]) OR CTLA-4 Inhibitors[Title/Abstract]) OR CTLA 4 Inhibitors[Title/Abstract]) OR Cytotoxic T-Lymphocyte-Associated Protein 4 Inhibitor[Title/Abstract]) OR Cytotoxic T Lymphocyte Associated Protein 4 Inhibitor[Title/Abstract]) OR CTLA-4 Inhibitor[Title/Abstract]) OR CTLA 4 Inhibitor[Title/Abstract]) OR PD-1 Inhibitor[Title/Abstract]) OR PD 1 Inhibitor[Title/Abstract]) OR Programmed Cell Death Protein 1 Inhibitor[Title/Abstract]) OR Immune Checkpoint Blockade[Title/Abstract]) OR Immune Checkpoint Inhibition[Title/Abstract]) OR PD-L1 Inhibitor[Title/Abstract]) OR PD L1 Inhibitor[Title/Abstract]) OR Programmed Death-Ligand 1 Inhibitors[Title/Abstract]) OR Programmed Death Ligand 1 Inhibitors[Title/Abstract]) OR PD-1-PD-L1 Blockade[Title/Abstract]) OR PD 1 PD L1 Blockade[Title/Abstract]
#8	#5 OR #6 OR #7
#9	“Chemoradiotherapy”[Mesh]
#10	(((((((((((((Chemoradiotherapies) OR Radiochemotherapy) OR Radiochemotherapies) OR Concurrent Chemoradiotherapy) OR Concurrent Chemoradiotherapies) OR Concomitant Chemoradiotherapy) OR Concomitant Chemoradiotherapies) OR Concomitant Radiochemotherapy) OR Concomitant Radiochemotherapies) OR Concurrent Radiochemotherapy) OR Concurrent Radiochemotherapies) OR Synchronous Chemoradiotherapy) OR Synchronous Chemoradiotherapies)
#11	#9 OR #10
#12	#3 AND #8 AND #11
**(b) Search strategy in Embase**	
#	Query
#1	‘non small cell lung cancer’/exp
#2	‘bronchial non small cell cancer’:ab,ti OR ‘bronchial non small cell carcinoma’:ab,ti OR ‘carcinoma, non-small-cell lung’:ab,ti OR ‘lung cancer, non small cell’:ab,ti OR ‘lung non small cell cancer’:ab,ti OR ‘lung non small cell carcinoma’:ab,ti OR ‘non oat cell lung cancer’:ab,ti OR ‘non small cell bronchial cancer’:ab,ti OR ‘non small cell cancer, lung’:ab,ti OR ‘non small cell lung carcinoma’:ab,ti OR ‘non small cell pulmonary cancer’:ab,ti OR ‘non small cell pulmonary carcinoma’:ab,ti OR ‘non squamous NSCLC’:ab,ti OR ‘non-oat cell lung cancer’:ab,ti OR ‘non-small-cell lung carcinoma’:ab,ti OR ‘nonsmall cell carcinoma of the lung’:ab,ti OR ‘nonsmall cell lung cancer’:ab,ti OR ‘nonsmall cell lung carcinoma’:ab,ti OR ‘pulmonary non small cell cancer’:ab,ti OR ‘pulmonary non small cell carcinoma’:ab,ti OR ‘non small cell lung cancer’:ab,ti
#3	#1 OR #2
#4	‘immune checkpoint inhibitor’/exp
#5	‘immune checkpoint inhibitor’/exp OR ‘Immune Checkpoint Blockers’:ab,kw,ti OR ‘Immune Checkpoint Inhibitor’:ab,kw,ti OR ‘CTLA-4 Inhibitors’:ab,kw,ti OR ‘CTLA 4 Inhibitors’:ab,kw,ti OR ‘Cytotoxic T-Lymphocyte-Associated Protein 4 Inhibitor’:ab,kw,ti OR ‘Cytotoxic T Lymphocyte Associated Protein 4 Inhibitor’:ab,kw,ti OR ‘CTLA-4 Inhibitor’:ab,kw,ti OR ‘CTLA 4 Inhibitor’:ab,kw,ti OR ‘PD-1 Inhibitor’:ab,kw,ti OR ‘PD 1 Inhibitor’:ab,kw,ti OR ‘Programmed Cell Death Protein 1 Inhibitor’:ab,kw,ti OR ‘Immune Checkpoint Blockade’:ab,kw,ti OR ‘Immune Checkpoint Inhibition’:ab,kw,ti OR ‘PD-L1 Inhibitor’:ab,kw,ti OR ‘PD L1 Inhibitor’:ab,kw,ti OR ‘Programmed Death-Ligand 1 Inhibitors’:ab,kw,ti OR ‘Programmed Death Ligand 1 Inhibitors’:ab,kw,ti OR ‘PD-1-PD-L1 Blockade’:ab,kw,ti OR ‘PD 1 PD L1 Blockade’:ab,kw,ti
#6	‘checkpoint inhibitor therapy’/exp
#7	‘check point blocking therapy’:ab,ti OR ‘check point inhibition therapy’:ab,ti OR ‘check point inhibitor therapy’:ab,ti OR ‘check point inhibitor-based therapy’:ab,ti OR ‘check point inhibitorbased therapy’:ab,ti OR ‘check point inhibitors therapy’:ab,ti OR ‘checkpoint blockade antibody therapy’:ab,ti OR ‘checkpoint blockade immune therapy’:ab,ti OR ‘checkpoint blockade immunotherapy’:ab,ti OR ‘checkpoint blockade therapy’:ab,ti OR ‘checkpoint blockage therapy’:ab,ti OR ‘checkpoint blocker immune therapy’:ab,ti OR ‘checkpoint blocker therapy’:ab,ti OR ‘checkpoint blocking antibody therapy’:ab,ti OR ‘checkpoint blocking immunotherapy’:ab,ti OR ‘checkpoint blocking therapy’:ab,ti OR ‘checkpoint immune therapy’:ab,ti OR ‘checkpoint immunotherapy’:ab,ti OR ‘checkpoint inhibition therapy’:ab,ti OR ‘checkpoint inhibitor antibody therapy’:ab,ti OR ‘checkpoint inhibitors therapy’:ab,ti OR ‘checkpoint inhibitory therapy’:ab,ti OR ‘immune checkpoint blockade therapy’:ab,ti OR ‘immune checkpoint blockage therapy’:ab,ti OR ‘immune checkpoint blocker therapy’:ab,ti OR ‘immune checkpoint blocking therapy’:ab,ti OR ‘immune checkpoint inhibition therapy’:ab,ti OR ‘immune checkpoint inhibitor therapy’:ab,ti OR ‘immune checkpoint mAb therapy’:ab,ti OR ‘immune checkpoint therapy’:ab,ti OR ‘immuno-checkpoint therapy’:ab,ti OR ‘immunocheckpoint therapy’:ab,ti OR ‘immunological checkpoint therapy’:ab,ti OR ‘inhibitor checkpoint therapy’:ab,ti OR ‘checkpoint inhibitor therapy’:ab,ti
#8	#5 OR #6 OR #7
#9	‘chemoradiotherapy‘/exp
#10	‘Chemoradiotherapies’:ab,kw,ti OR ‘Radiochemotherapy’:ab,kw,ti OR ‘Radiochemotherapies’:ab,kw,ti OR ‘Concurrent Chemoradiotherapy’:ab,kw,ti OR ‘Concurrent Chemoradiotherapies’:ab,kw,ti OR ‘Concomitant Chemoradiotherapy’:ab,kw,ti OR ‘Concomitant Chemoradiotherapies’:ab,kw,ti OR ‘Concomitant Radiochemotherapy’:ab,kw,ti OR ‘Concomitant Radiochemotherapies’:ab,kw,ti OR ‘Concurrent Radiochemotherapy’:ab,kw,ti OR ‘Concurrent Radiochemotherapies’:ab,kw,ti OR ‘Synchronous Chemoradiotherapy’:ab,kw,ti OR ‘Synchronous Chemoradiotherapies’:ab,kw,ti
#11	#9 OR #10
#12	#3 AND #8 AND #11
**(c) Search strategy in Web of Science**	
#	Query
#1	TS = (non small cell lung cancer OR Non Small Cell Lung OR Non-Small-Cell Lung OR Non-Small-Cell Lung Carcinomas OR Non-Small Cell Lung Cancer OR Non-Small-Cell Lung Carcinoma OR Non Small Cell Lung Carcinoma OR Nonsmall Cell Lung Cancer OR Non-Small Cell Lung Carcinoma)
#2	TS = (cancer immunotherapy OR immunotherapy, cancer OR tumor immunotherapy OR tumour immunotherapy OR cancer immunotherapy OR checkpoint inhibitor therapy OR check point blocking therapy OR check point inhibition therapy OR check point inhibitor therapy OR check point inhibitor-based therapy OR check point inhibitorbased therapy OR check point inhibitors therapy OR checkpoint blockade antibody therapy OR checkpoint blockade immune therapy OR checkpoint blockade immunotherapy OR checkpoint blockade therapy OR checkpoint blockage therapy OR checkpoint blocker immune therapy OR checkpoint blocker therapy OR checkpoint blocking antibody therapy OR checkpoint blocking immunotherapy OR checkpoint blocking therapy OR checkpoint immune therapy OR checkpoint immunotherapy OR checkpoint inhibition therapy OR checkpoint inhibitor antibody therapy OR checkpoint inhibitors therapy OR checkpoint inhibitory therapy OR immune checkpoint blockade therapy OR immune checkpoint blockage therapy OR immune checkpoint blocker therapy OR immune checkpoint blocking therapy OR immune checkpoint inhibition therapy OR immune checkpoint inhibitor therapy OR immune checkpoint mAb therapy OR immune checkpoint therapy OR immuno-checkpoint therapy OR immunocheckpoint therapy OR immunological checkpoint therapy OR inhibitor checkpoint therapy OR checkpoint inhibitor therapy)
#3	TS = (chemoradiotherapy OR Chemoradiotherapies OR Radiochemotherapy OR Radiochemotherapies OR Concurrent Chemoradiotherapy OR Concurrent Chemoradiotherapies OR Concomitant Chemoradiotherapy OR Concomitant Chemoradiotherapies OR Concomitant Radiochemotherapy OR Concomitant Radiochemotherapies OR Concurrent Radiochemotherapy OR Concurrent Radiochemotherapies OR Synchronous Chemoradiotherapy OR Synchronous Chemoradiotherapies)
#4	#1 AND #2 AND #3
**(d) Search strategy in ClinicalTrials**	
Condition/disease	NSCLC, Stage III
Other terms	Chemoradiotherapy
Intervention/treatment	Immunotherapy

**Table A2:** Inclusion and exclusion criteria for records in the Meta-analysis.

**(A) The inclusion criteria:**
(1) Prospective or retrospective studies that conduct clinicopathological examinations of stage III unresectable NSCLC; (2) Treated with concurrent chemoradiotherapy and immunotherapy; (3) Reported the incidence of RP; (4) Published in English.
**(B) The exclusion criteria:**
(1) Studies reported only patient survival data (e.g., OS, PFS) without the occurrence of RP; (2) Use of sequential chemoradiotherapy; (3) Use of radiotherapy alone; (4) Immunotherapy not administered; (5) Inclusion of other tumor types (e.g., small-cell lung and esophageal cancers); (6) Inclusion of patients with non-stage III NSCLC; (7) Non-investigational articles (e.g., abstracts, letters, tumor reviews, and systematic reviews); (8) Articles on other topics (e.g., animal and molecular experiments).

**Table A3:** Inclusion and exclusion criteria for patients in the real-world retrospective study.

**(A) The inclusion criteria:**
(1) Pathologically and clinically confirmed diagnosis of stage III unresectable NSCLC per the 9th edition of AJCC staging; (2) Treatment with cCRT and immunotherapy using anti-PD-1/anti-PD-L1; (3) Age ≥18 years; (4) Availability of complete clinical, imaging, and radiotherapy planning data; (5) ECOG PS of ≤1, with no significant dysfunction in cardiac, hepatic, renal, or other major organs; (6) Total radiotherapy dose of ≥40 Gy; (7) Follow-up duration of >6 months.
**(B) The exclusion criteria:**
(1) History of prior chest radiotherapy; (2) Severe lung infection; (3) Sequential radiotherapy; (4) Prior surgery; (5) Incomplete clinical data.

**Table A4:** The quality assessment of the included non-RCT studies.

Author (Year)	Selection				Comparability	Outcome			Score
	A	B	C	D	E	F	G	H	
Lukas Käsmann (2023) [16]	1	1	1	1	1	1	1	1	8
Romana Wass (2022) [18]	1	1	1	1	2	1	1	1	9
Jonathan Q Trinh (2024) [23]	1	1	1	1	2	1	1	1	9
Adrien Holzgreve (2024) [24]	1	1	1	1	2	1	1	1	9
Mizuki Sato (2024) [25]	1	1	1	1	2	1	1	1	9
Donna M Edwards (2024) [26]	1	1	1	1	1	1	1	1	8
Luqing Zhao (2024) [29]	1	1	1	1	2	1	1	1	9
Song Guan (2024) [34]	1	1	1	1	1	1	1	1	8
Song Guan (2023) [35]	1	1	1	1	2	1	1	1	9

NOS criteria: Selection (A: representativeness of cases, B: selection of controls, C: exposure ascertainment, and D: no death when investigation begins); Comparability (E: comparable on confounders); Outcome (F: outcome assessment, G: adequate follow-up, and H: loss to follow-up rate).
