# Supplementary figures and images for: Early Immune Checkpoint Inhibitor Administration Increases the Risk of Radiation-Induced Pneumonitis in Patients with Stage III Unresectable NSCLC Undergoing Chemoradiotherapy
Source: Cancers (Basel). 2025 May 20;17(10):1711. doi: 10.3390/cancers17101711 (PMC12110373; doi:10.3390/cancers17101711)

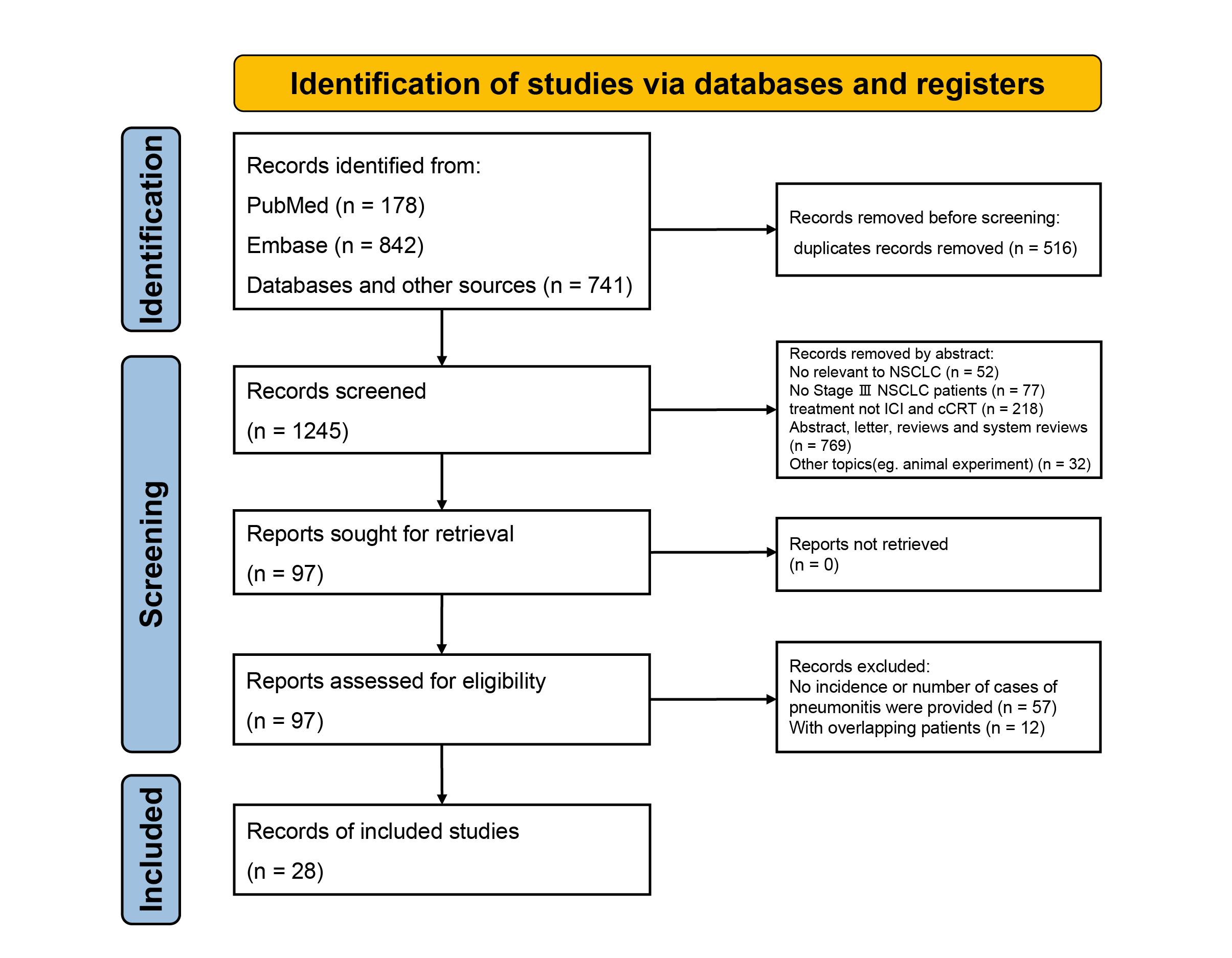

Supplement: Supplementary file 1 [file cancers-17-01711-s001.zip › Figure S1.tif]

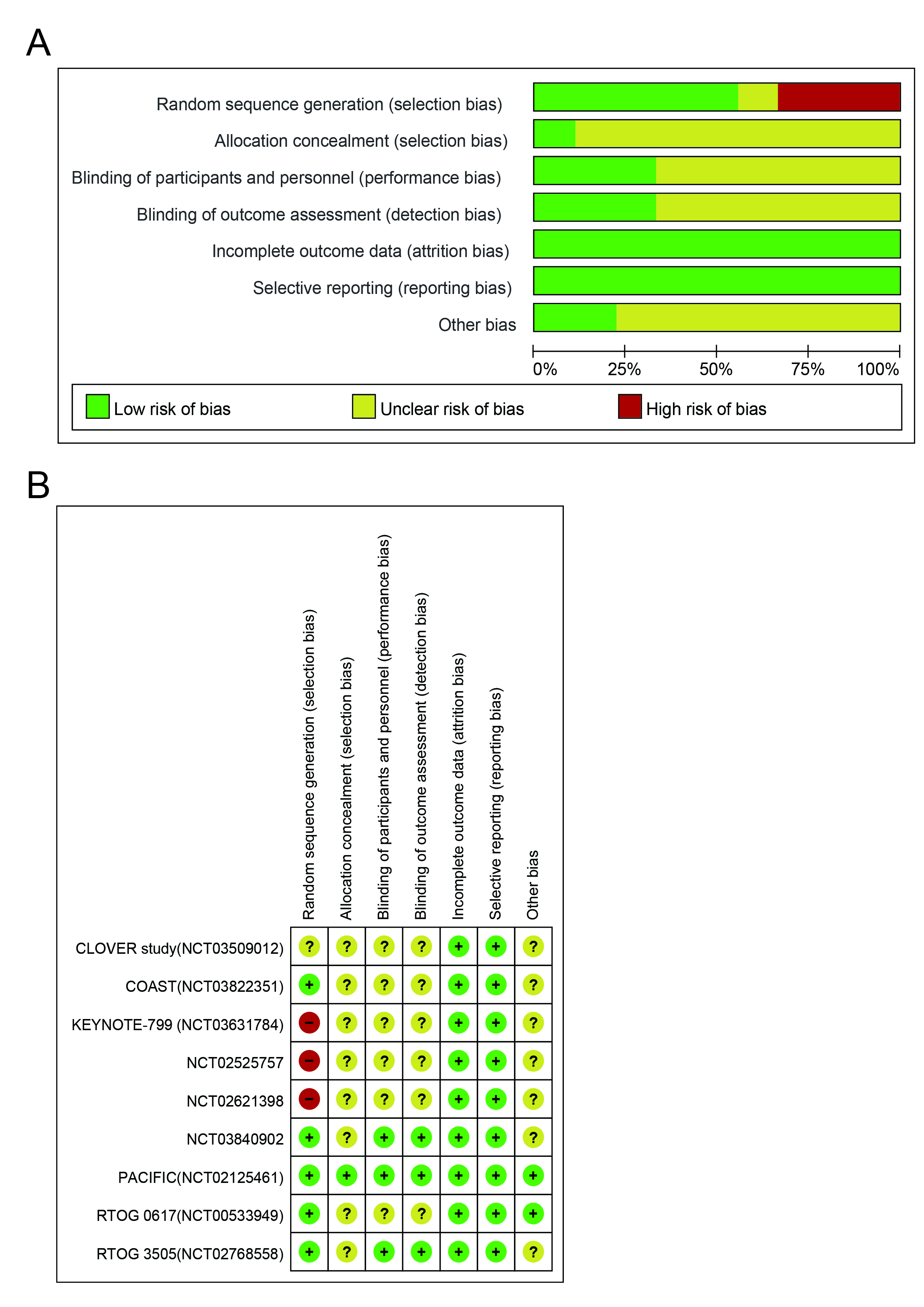

Supplement: Supplementary file 1 [file cancers-17-01711-s001.zip › Figure S2.tif]

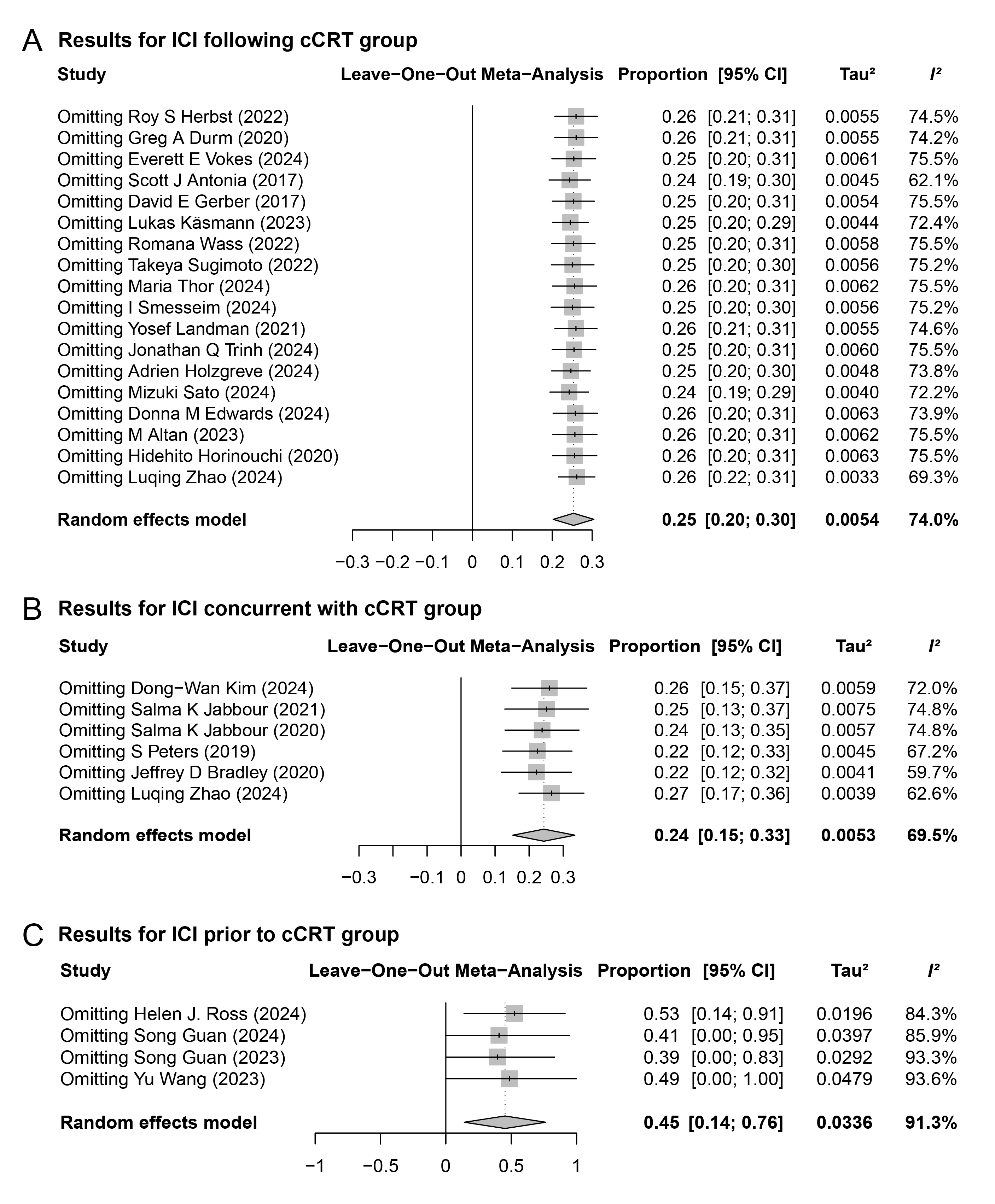

Supplement: Supplementary file 1 [file cancers-17-01711-s001.zip › Figure S3.tif]

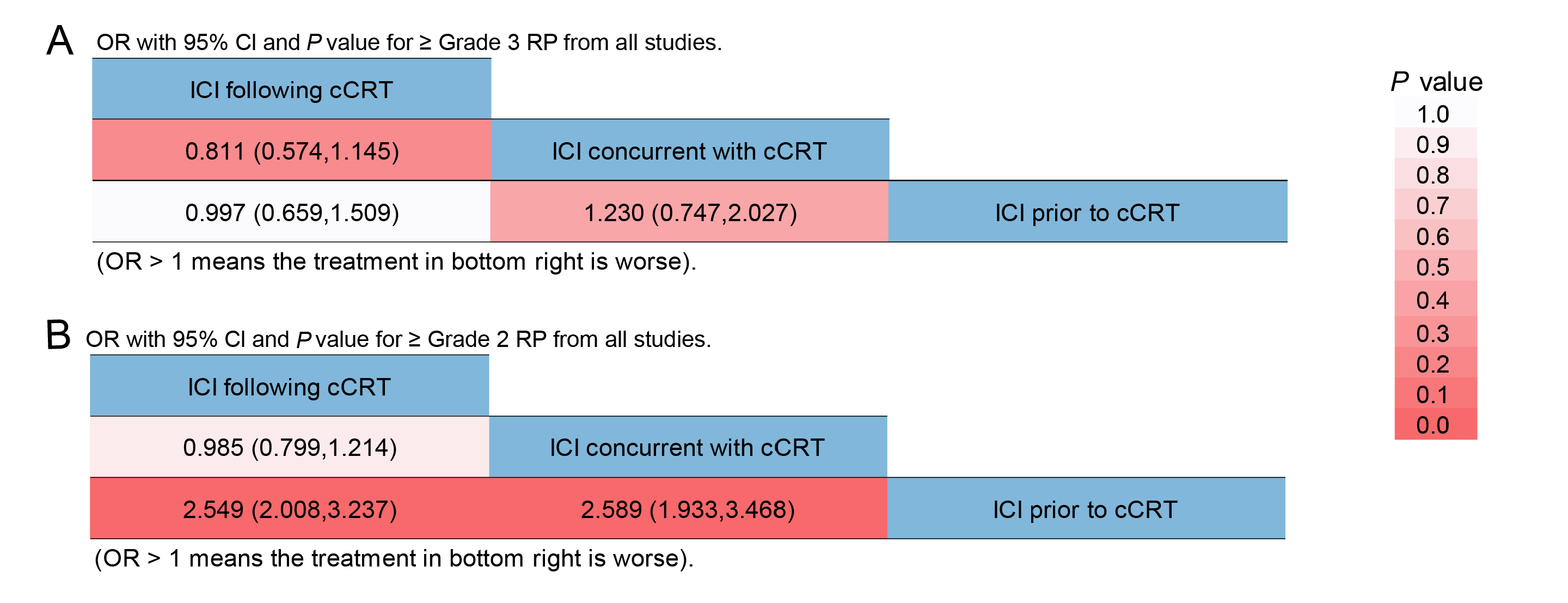

Supplement: Supplementary file 1 [file cancers-17-01711-s001.zip › Figure S4.tif]

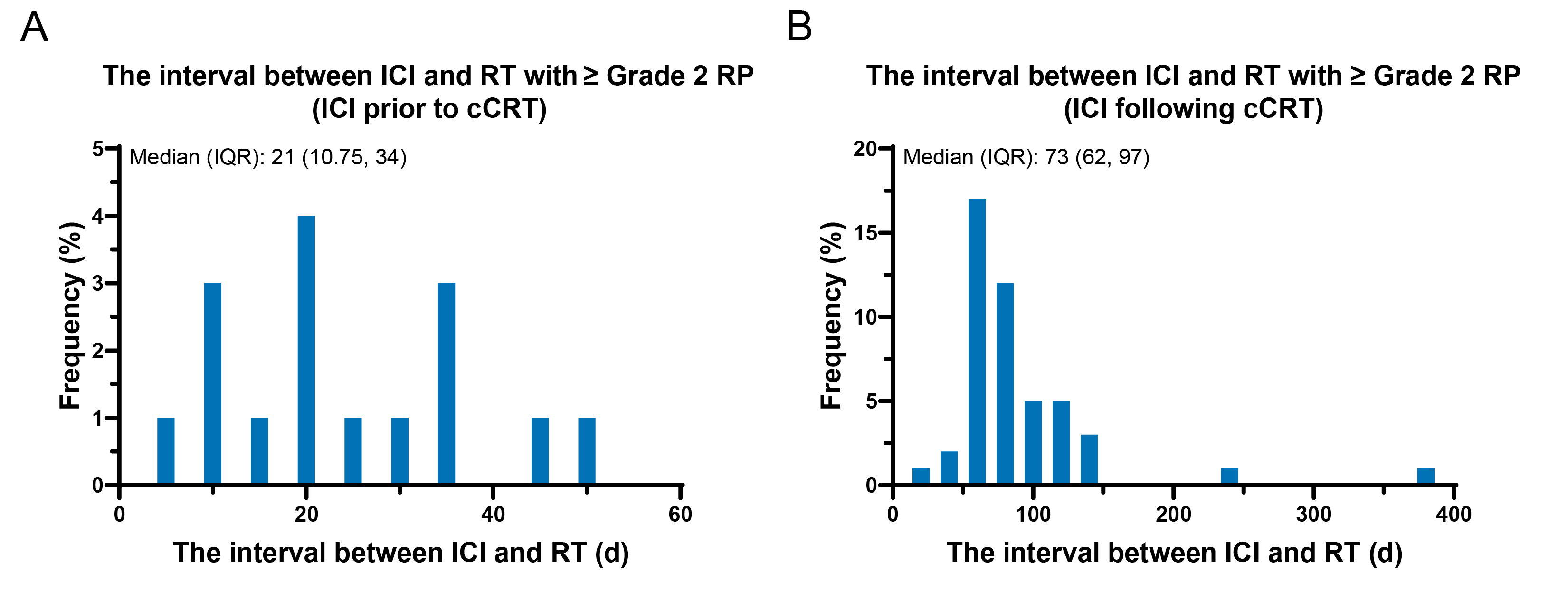

Supplement: Supplementary file 1 [file cancers-17-01711-s001.zip › Figure S5.tif]
